# Molecular Mechanisms of Nanomaterial-Bacterial Interactions Revealed by Omics—The Role of Nanomaterial Effect Level

**DOI:** 10.3389/fbioe.2021.683520

**Published:** 2021-06-14

**Authors:** Monika Mortimer, Ying Wang, Patricia A. Holden

**Affiliations:** ^1^Institute of Environmental and Health Sciences, College of Quality and Safety Engineering, China Jiliang University, Hangzhou, China; ^2^Environmental Genomics and Systems Biology Division, Lawrence Berkeley National Laboratory, Berkeley, CA, United States; ^3^Bren School of Environmental Science and Management and Earth Research Institute, University of California, Santa Barbara, Santa Barbara, CA, United States

**Keywords:** engineered nanomaterials, bacteria, pathways, transcriptomic, proteomic, metabolomic

## Abstract

Nanotechnology is employed across a wide range of antibacterial applications in clinical settings, food, pharmaceutical and textile industries, water treatment and consumer goods. Depending on type and concentration, engineered nanomaterials (ENMs) can also benefit bacteria in myriad contexts including within the human body, in biotechnology, environmental bioremediation, wastewater treatment, and agriculture. However, to realize the full potential of nanotechnology across broad applications, it is necessary to understand conditions and mechanisms of detrimental or beneficial effects of ENMs to bacteria. To study ENM effects, bacterial population growth or viability are commonly assessed. However, such endpoints alone may be insufficiently sensitive to fully probe ENM effects on bacterial physiology. To reveal more thoroughly how bacteria respond to ENMs, molecular-level omics methods such as transcriptomics, proteomics, and metabolomics are required. Because omics methods are increasingly utilized, a body of literature exists from which to synthesize state-of-the-art knowledge. Here we review relevant literature regarding ENM impacts on bacterial cellular pathways obtained by transcriptomic, proteomic, and metabolomic analyses across three growth and viability effect levels: inhibitory, sub-inhibitory or stimulatory. As indicated by our analysis, a wider range of pathways are affected in bacteria at sub-inhibitory vs. inhibitory ENM effect levels, underscoring the importance of ENM exposure concentration in elucidating ENM mechanisms of action and interpreting omics results. In addition, challenges and future research directions of applying omics approaches in studying bacterial-ENM interactions are discussed.

## Introduction

Understanding interactions of engineered nanomaterials [ENMs, materials with at least one dimension ≤100 nm and having unique size-related physico-chemical properties (Hochella et al., 2019)] with bacteria is important for several reasons. First, there is the need to guide the design and use of ENMs in antibacterial applications, driven by global issues including antibiotic resistance and drinking water scarcity (Mauter et al., 2018; Cheeseman et al., 2020; Huang et al., 2020). Second, risk assessment of ENM exposures to environmental and beneficial bacteria may be of interest (Holden et al., 2013, 2014b). Lastly, as an emerging trend, ENMs can stimulate beneficial bacterial functions (Mu et al., 2019; Peng et al., 2019; Ren et al., 2019b; Yan et al., 2019). Successful accomplishment of these aims requires detailed knowledge of the underlying mechanisms of ENM-bacterial interactions.

Over the past 15 years, myriad studies have been conducted regarding ENM effects to bacteria, providing valuable information on ENM mechanisms of action. However, most studies to date have used targeted assays which are limited to assessing just a few endpoints out of hundreds of metabolic pathways in bacterial cells (Le Boulch et al., 2019). To overcome this limitation, omics (e.g., transcriptomics, proteomics, and metabolomics) methods have been increasingly employed for non-targeted analyses of ENM effects (Revel et al., 2017).

Non-targeted assessment of differential gene expression, protein or metabolite levels in organisms exposed to ENMs, combined with pathway analysis, allows for measuring global responses to ENMs at the molecular level and can reveal subtle changes in cellular and organismal physiology that are not identifiable using conventional assays that measure endpoints such as growth, viability, oxidative stress, or membrane damage. Progress in genome sequencing, mass spectroscopy, bioinformatics and correlating genome annotations with functional information has enabled implementation of omics methods in environmental toxicology, including nanotoxicology (Costa and Fadeel, 2016). Advantages of omics techniques in nanotoxicology have been reviewed from human and environmental safety and risk assessment perspectives (Costa and Fadeel, 2016; Fröhlich, 2017; Revel et al., 2017; Fadeel et al., 2018; Majumdar and Keller, 2020), but a systematic overview of recent progress in omics methods used for elucidating ENM effects on bacteria is lacking. Due to the differences in cell structure and physiology of eukaryotic and prokaryotic organisms, mechanisms of interactions between bacteria and ENMs are expected to be different from those between animal or plant cells and ENMs (Wang et al., 2016, 2018a; Westmeier et al., 2018), and thus need special scrutiny.

ENM exposure concentration and other test conditions are crucial when assessing ENM biological effects (Holden et al., 2016). It has been demonstrated that, in many published studies, ENM hazard assessment has been performed at higher ENM concentrations than predicted to occur in the environment (Holden et al., 2014a). In hazard assessment, ENM concentrations and other test conditions should be chosen to address specific research questions for realistic exposure scenarios, thereby defining “environmental relevance” situationally (Holden et al., 2016). For example, different areas of research regarding bacterial-ENM interactions, such as (i) antibacterial mechanisms, (ii) hazards to environmental and beneficial bacteria, and (iii) ENM-enabled enhancement of bacterial beneficial functions, require exposures to ENMs at concentrations which induce three different levels of effects on bacterial growth or viability: inhibitory, sub-inhibitory, or stimulatory. “Sub-inhibitory” ENM concentrations have been defined here as ENM concentrations which induce no growth inhibition or loss of viability in bacteria. Since bacterial metabolic activities and viability differ markedly across these three thresholds, varying molecular responses to ENM exposures are evidenced in different omics studies. Given the growing body of literature in omics applied to bacterial-ENM interactions, a synthesis of lessons learned is needed to guide future work.

Although ENM effects on bacteria depend on several variables (e.g., exposure time, temperature, light intensity, and media composition), ENM exposure concentration is the variable which is commonly selected for omics studies based on preliminary dose-response assessments, and thus can be considered an important factor in determining the ENM effect levels. Here, in this review, we ask: what are the relationships between ENM effect level and bacterial metabolic pathway effects? Across various ENMs, what metabolic pathway responses are associated with inhibitory, sub-inhibitory and stimulatory effects on bacterial growth or viability? How does choice of ENM concentration causing different effect levels affect understanding bacterial responses to ENMs? To address these questions, we review published literature regarding ENM mechanisms of action in bacteria obtained by transcriptomic, proteomic, and metabolomic analyses. We synthesize data regarding ENM-induced changes in metabolic pathways and cellular processes across different ENM types and effect levels. We compare the reported molecular responses to ENMs in bacteria for the three “ENM effect” categories and discuss the challenges and opportunities of omics approaches in studying bacterial-ENM interactions. Our work provides a secondary analysis of published results such that, across various ENMs and bacterial taxa, new insights are gained regarding what bacterial response mechanisms constitute observable population-level outcomes, such as differences in growth rate, yield, or death rate. At ENM concentrations that appear sub-inhibitory to bacterial growth, for example, significant molecular activity remodeling may still be occurring which allows for apparent compensation at the population level. The understanding of molecular mechanistic responses to ENMs would logically, therefore, relate to ENM exposure concentration. This review intends to stimulate thinking and discussion on the significance of ENM exposure concentrations and effect levels, with aims of assisting future hazard assessment experimental designs and of reconciling future omics analysis results with expected bacterial physiological states and processes.

## Methods

A literature search via the Web of Science (Clarivate Analytics) was conducted in August 2020, using key words “nanoparticle^∗^ OR nanomaterial^∗^” AND “bacteria” AND “transcriptomic^∗^ OR proteomic^∗^ OR metabolom^∗^.” From each reviewed article, data regarding the type and method of omics approach, number of biological replicates and statistical criteria for differential regulation of genes, proteins or metabolites, bacterial strain, ENM type, size, surface charge, and functionalization, tested ENM concentrations, exposure duration, and physiological effects at the tested ENM concentrations were extracted and recorded in a spreadsheet (Supplementary Table 1). In addition, major findings of each paper were summarized and included in the spreadsheet. Papers were first categorized based on the type of omics approach (column B in Supplementary Table 1). Within each omics category, the papers were further categorized into “inhibitory,” “sub-inhibitory,” and “stimulatory” effect levels (column F in Supplementary Table 1) based on the reported effects on bacterial growth or viability (column N “Physiological effects at tested NP conc.” in Supplementary Table 1). Namely, the studies where the ENM concentration selected for the omics assay caused growth inhibition or lethality in bacteria were labeled “inhibitory”; studies where omics assays were conducted using ENM concentrations which did not significantly diminish bacterial growth or viability were labeled as “sub-inhibitory”; and studies where omics assays were performed with bacteria exposed to ENMs at concentrations which stimulated bacterial growth or a beneficial function, were termed “stimulatory.” Among analyzed papers, only three papers focused on other physiological effects than growth or viability: one paper reported chlorophyll a (Chl-a) reduction, another paper decreased nitrate removal efficiency and the third paper increased nitrogen removal rate upon ENM exposures; so they were categorized into “inhibitory,” “inhibitory,” and “stimulatory” effect levels, respectively.

Omics results were extracted from the papers and recorded (Supplementary Table 2). Specifically, the total numbers of affected genes, proteins or metabolites, as stated in the papers, were recorded in a spreadsheet (row 5 in Supplementary Table 2). The values were used for generating a box plot to compare the numbers of affected genes in ENM-exposed bacteria at two different physiological effect levels (inhibitory and sub-inhibitory). Significant differences were determined by two-tailed Student’s *t*-test (*p* < 0.05). For Kyoto Encyclopedia of Genes and Genomes (KEGG)^1^ pathway analysis, data regarding the affected KEGG pathways were extracted from the papers. If KEGG pathways were not identified in the original publication, the differentially expressed genes or proteins listed in the publication were assigned to a KEGG pathway. The affected pathways were not analyzed based on the down- or upregulation by ENMs, because not all studies had conclusive results on the direction of the whole pathway regulation (i.e., some genes/proteins/metabolites in a pathway may have been downregulated and some upregulated). Thus, only “affected” pathways were identified and analyzed here. First, the affected KEGG pathway analysis was conducted by ENM type by compiling affected KEGG pathways for each ENM type, including data from multiple articles if the ENM type was studied in more than one paper. Second, the affected KEGG pathways were analyzed by the categories of the physiological effect level (inhibitory, sub-inhibitory, and stimulatory) to determine the frequency of reporting each pathway at each of the three ENM effect levels. The numbers of papers reporting the affected pathway, regardless of the ENM type, were counted in each of the three categories.

## Results

### Quantity and Attributes of the Retrieved Literature

The literature search yielded 41 publications reporting using omics techniques for measuring ENM-induced effects in bacteria (Supplementary Table 1), of which 39 reported significant changes in bacterial metabolic pathways or cellular processes upon exposure to ENMs, and thus were included in the analysis presented herein. All articles regarded a single bacterial strain, except one. For the article that included results for two bacterial strains, the results were analyzed as two separate studies, resulting in a total of 40 studies analyzed (Supplementary Table 2). More than half of the total 41 retrieved papers were published between 2018 and 2020, which illustrates the recent trend of implementing omics techniques in bacterial nanotoxicology. Among these papers, transcriptomics and proteomics were the most prevalently used methods (17 proteomics papers and 15 transcriptomics papers) while metabolomics was the least employed (3 papers). Additionally, some studies used transcriptomics in combination with either proteomics (4 papers) or metabolomics (1 paper); one study employed proteomics and metabolomics. Among the studies using transcriptomics, 60% employed RNA sequencing (RNAseq) as a method of choice while the rest used a microarray-based approach (Supplementary Table 1). Regardless of the method, a twofold change in the gene transcription level was set as the significance threshold in the majority of the studies. Most of the transcriptomics studies included 3 biological replicates (range: 2–8), while one paper reported no information about replicates. Half of the proteomics studies reviewed applied 2D-gel electrophoresis-based protein quantification, followed by identification of proteins or peptides using liquid chromatography tandem mass spectroscopy (LC-MS/MS). The rest of the studies conducted quantitative untargeted proteome analysis using online nano-flow or micro-flow LC-MS/MS techniques either with or without isobaric labeling; one study used an emerging data-independent method SWATH-MS (Sequential Window Acquisition of all Theoretical Mass Spectra). The number of biological replicates in proteomics studies ranged between 2 and 10, with one study reporting no information on replicates. Among the five metabolomics studies, three used nuclear magnetic resonance (NMR)-based techniques, two LC-MS, and one gas chromatography (GC)-MS-based non-targeted metabolomics, while including 3-6 biological replicates. Overall, most studies included in the current analysis used at least three biological replicates and reported the criteria for statistical analysis. Since the aim of the current review was to give an overview and synthesize the pathway-related data reported in relevant peer-reviewed literature, we excluded studies which did not identify changes in the biological pathways in response to ENM exposures (column P in Supplementary Table 1).

### Overview of Bacterial Pathways Affected by ENMs

**FIGURE 1:**
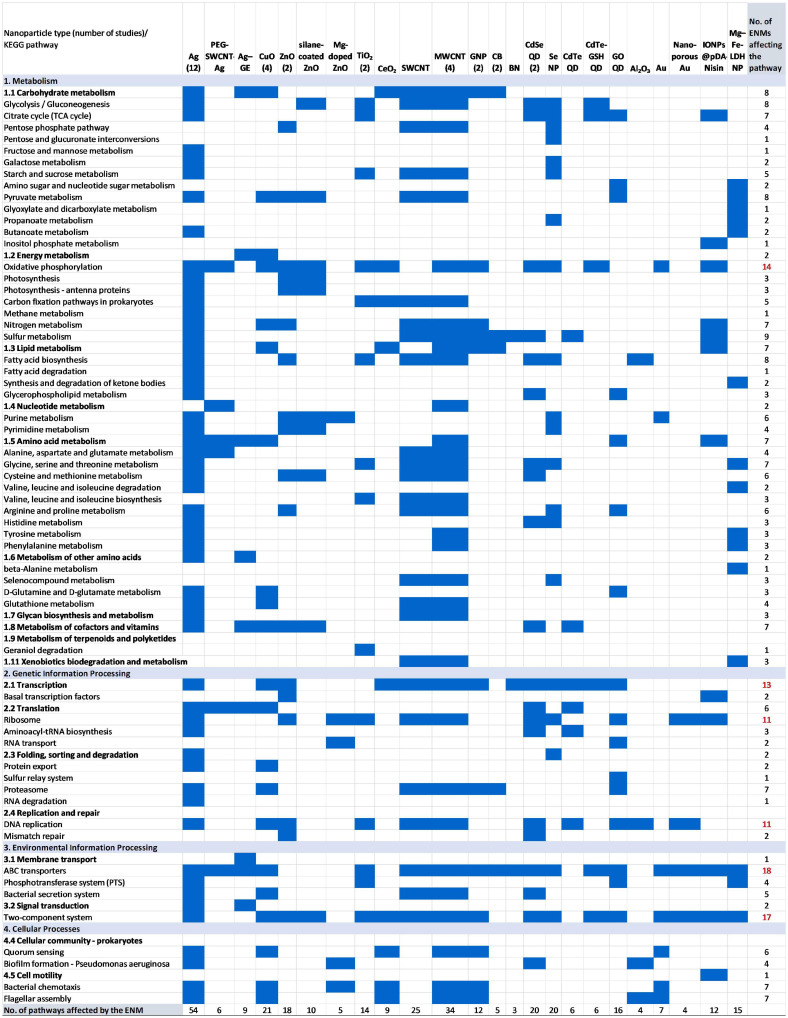
Affected KEGG pathways in bacteria upon exposure to engineered nanomaterials (ENMs) based on 40 published studies using omics analyses (Supplementary Table 2). Forty studies resulted in 46 entries since some papers regarded more than one ENM type. If the ENM was assessed in more than one study, the number of papers is indicated in parentheses after the ENM type. For KEGG pathways that were affected by more than 10 ENMs, the number of ENMs is indicated in red (rightmost column). PEG-SWCNT-Ag, pegylated Ag-coated single-wall carbon nanotubes; Ag-GE, Ag nanoparticle–graphene nanocomposites; MWCNTs, multiwall carbon nanotubes; GNP, graphene platelets; CB, carbon black; BN, boron nitride; CdSe QD, CdSe quantum dots; CdTe GSH QD, CdTe glutathione quantum dots; IONPs@pDA Nisin, nisin-loaded iron oxide nanoparticle polydopamine composites; Mg–Fe-LDH NPs, Mg–Fe layered double hydroxide nanoparticles.

In total, at least 24 different types of ENMs (not considering different commonly used coatings of Ag nanoparticles (NPs), such as citrate and polyvinylpyrrolidone) had been studied for their effects on bacteria using omics assays (Figure 1). Among these, Ag NPs were by far the most studied ENM type (in 12 papers). This is expected, considering the antibacterial properties of Ag^+^ and Ag NPs, and thus the high interest in exploring the mechanisms of action of nanosized Ag due to its applicability in consumer products and in medical fields. Two other common antibacterial ENMs—CuO and ZnO NPs—had been studied in 4 and 2 papers, respectively. Other ENMs which were examined in more than one paper regarding bacteria and omics techniques included multiwall carbon nanotubes (MWCNTs, 4 papers), TiO_2_ nanoparticles (2 papers), graphene nanoplatelets (GNP, 2 papers), carbon black (CB, 2 papers), and CdSe quantum dots (QDs, 2 papers). The characteristics of ENMs along with the references have been listed in Supplementary Table 1.

From all of the KEGG pathways that were affected by ENMs, regardless of the physiological effect, membrane transport (ABC transporters), signal transduction (two-component systems), energy metabolism (oxidative phosphorylation), transcription, translation and DNA replication were disturbed by more than 10 different types of ENMs (Figure 1). Among these, the membrane-associated pathways are expected to be affected in ENM-exposed bacteria because of the established importance of physical contact between ENMs and bacteria in inducing biological effects (Hakamada et al., 2017; Mortimer et al., 2020). Many essential functions such as signal transduction, import and export of nutrients and metabolites, as well as respiratory chain components, are all located in the membrane of bacteria. Thus, differently from eukaryotic cells where many cellular functions are carried out in designated intracellular organelles, and often cellular uptake of ENMs is necessary for biological effects, bacteria are susceptible to ENMs without cellular uptake. The latter has been demonstrated with effective antibacterial surfaces such as vertically aligned graphene oxide nanosheets (Lu et al., 2017), or pronounced transcriptomic changes induced by ENMs assembled into micrometer-size agglomerates, which could not be internalized by bacteria due to size restrictions (Mortimer et al., 2020). Interestingly, the commonly affected KEGG pathways in bacteria across all ENMs and bacterial strains (Figure 1) included the same major cellular functions as were identified in multicellular organisms by Burkard et al. (2020). Specifically, Burkard et al. (2020) conducted a meta-analysis of transcriptomic data of three environmental model organisms, *Arabidopsis thaliana*, *Caenorhabditis elegans*, and *Danio rerio*, exposed to different ENMs, and revealed that common gene expression patterns across different organisms and ENMs included energy generation, general signaling, and DNA metabolism (Burkard et al., 2020). These functional categories were indicative of mitochondrial- and DNA-related ENM effects and impaired signal transduction which are impacted in eukaryotic organisms upon cellular uptake of ENMs. Since these affected pathways were essential for the viability of the analyzed eukaryotic organisms, the omics findings expectedly confirmed the results of physiological assays (Burkard et al., 2020). An analysis of the prokaryotic pathway results conducted herein also indicated that the major affected pathways identified with omics techniques confirmed physiological assay findings: ENMs affect bacteria mainly *via* membrane damage, reactive oxygen species (ROS) generation and shedding of toxic metal ions that can contribute to excessive ROS and membrane damage. However, the additional value of omics methods manifests in revealing ENM-affected cellular functions that are not commonly measured in physiological assays. These pathways, regulated by at least six ENMs, included glycolysis/gluconeogenesis, the citrate cycle and pyruvate metabolism, nitrogen and sulfur metabolism, fatty acid biosynthesis, purine metabolism, amino acid (glycine, serine and threonine, cysteine and methionine, arginine and proline) metabolism, metabolism of cofactors and vitamins, proteasome, quorum sensing, chemotaxis, and flagellar assembly (Figure 1).

Regarding the number of pathways affected by specific ENM types, Ag topped the list with 54 dysregulated KEGG pathways (Figure 1). The value is likely biased due to the high number of studies conducted with Ag NPs compared to other types of ENMs. Excluding Ag NPs, other ENMs which have been shown to induce changes in at least 20 KEGG pathways are single-wall carbon nanotubes (SWCNTs), MWCNTs, CuO NPs, CdSe QDs, and Se NPs; boron nitride (BN), Al_2_O_3_, nanoporous Au, CB, and Mg-doped ZnO appear relatively less potent (Figure 1). Admittedly, this comparison does not take into account ENM effect levels in bacteria which can influence the numbers of genes and pathways affected (Mortimer et al., 2020). To shed light on this aspect, in the following sections we analyze the published omics data based on the three “physiological effect” exposure concentration categories: inhibitory, sub-inhibitory, and stimulatory ENM concentrations.

### Transcriptomic, Proteomic, and Metabolomic Responses Vary With ENM Effect Levels

From assessing the published literature, it is observed that ENM exposure concentrations used in mechanistic studies have been mainly driven by the aim of the study (Supplementary Table 1). Namely, inhibitory ENM concentrations appear to have been applied when the study purpose has been to elucidate antibacterial mechanisms of ENMs intended for eradicating bacteria. ENM doses which are not inhibitory to bacterial growth or viability have been applied when the purpose is to understand the mechanisms of interactions between bacteria and ENMs at exposure concentrations estimated to occur in the environment resulting either from incidental release or intentional application, e.g., low concentrations in agriculture. Further, a set of studies has emerged wherein ENMs have been found to exert beneficial effects on bacteria which could be utilized in environmental remediation. For these latter studies, omics techniques have been used to explore the underlying mechanisms of ENM stimulatory actions in bacteria.

**FIGURE 2:**
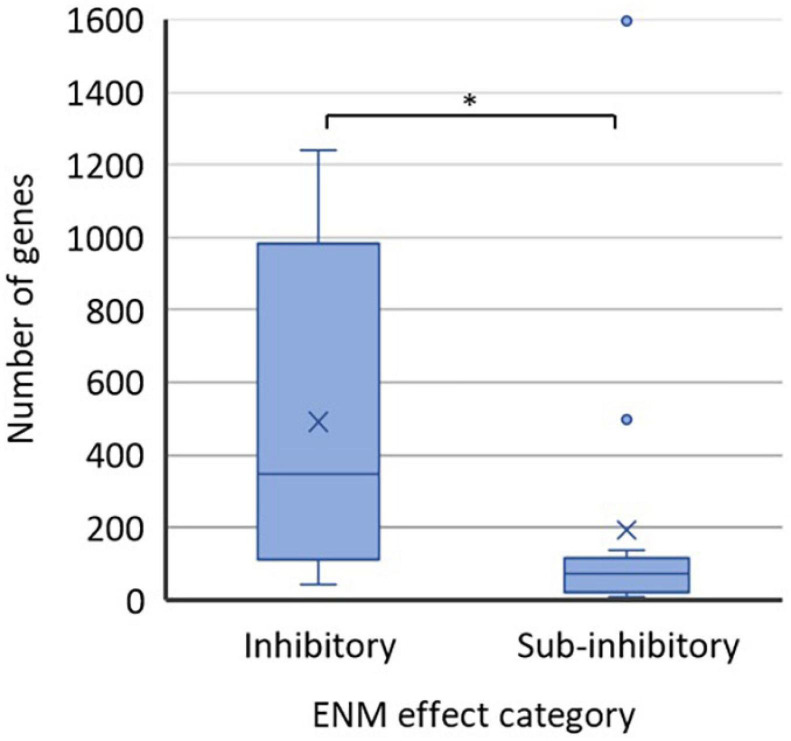
Numbers of affected genes in ENM-exposed bacteria at physiological effect levels of ENMs which were either inhibitory (12 data points) or sub-inhibitory (15 data points) to bacteria. Values were extracted from the published literature consulted herein (Supplementary Table 2). The studies employed either RNAseq or microarray-based approach and mostly used the significance threshold of twofold change in the gene transcription (Supplementary Table 1). The bottom lines of the boxes represent the 1st quartile and the top lines of the boxes represent the 3rd quartile; the middle lines of the boxes are the median and the **×** are the mean values of the datasets. The whiskers show the minimum and maximum values; the data points outside the range of whiskers are considered outliers. ^∗^*p* < 0.05, *t*-test.

It is a common perception that high ENM exposure concentrations which induce a detectable physiological response such as growth inhibition or loss of viability are accompanied by higher numbers of altered genes, proteins, or metabolites than sub-inhibitory ENM exposure levels. With the caveat that analytical techniques, data treatment protocols, and statistical criteria may vary between studies (Supplementary Table 1) we used published data on differentially regulated genes to shed light on this aspect. Transcriptomics-based data was used for the analysis because the coverage from transcriptomics analysis is inherently greater than from proteomics or metabolomics, providing the best metrics for global changes in bacteria. Additionally, most of the transcriptomics studies (using either RNAseq or microarray-based approach) used the same significance threshold (twofold change in the gene transcription). Thus, the comparison of the extent of biological response at different ENM effect levels was deemed appropriate based on the numbers of regulated genes (Figure 2). The results of the analysis supported the general perception, demonstrating a significantly higher number of genes being dysregulated at inhibitory ENM effect levels than at sub-inhibitory effect levels. However, in the category of “sub-inhibitory ENM effects” there were two papers that reported considerably higher numbers of affected genes [1,598 genes from RNAseq analysis (Mu et al., 2016) and 497 genes from microarray analysis (McQuillan and Shaw, 2014)] as compared to the rest of the 13 papers in this category. One of the two outliers (1,598 genes) exceeded the numbers of dysregulated genes in all studies using inhibitory ENM concentrations (Figure 2). Since the criteria for the statistical significance in identifying these genes as differentially expressed were not clear from the paper (Supplementary Table 1), the value should be treated critically. However, the value reported by McQuillan and Shaw, 2014 suggests that it would be incorrect to assume that there is a less pronounced omics response at sub-inhibitory, relative to inhibitory ENM concentrations.

#### Omics Studies With Bacteria at Inhibitory ENM Effect Levels

The majority of the reviewed studies that used omics approaches employed ENM concentrations causing bacterial physiological responses that were measurable with conventional endpoints such as growth inhibition and lethality, and assays such as membrane damage and oxidative stress (Supplementary Table 1). In these studies, the omics analyses often corroborated the physiological effects (e.g., induction of oxidative stress or inhibition of photosynthesis) and sometimes provided no additional insight into antibacterial mechanisms. However, some studies confirmed that conducting omics analysis upon exposure to ENMs at inhibitory concentrations is a useful tool for elucidating antibacterial mechanisms of novel antibacterial ENMs (Song et al., 2019). For example, antibacterial nisin-loaded iron oxide NPs (IONPs@pDA-Nisin) exerted unexpected transcriptional effects in a common food spoilage bacterium *Alicyclobacillus acidoterrestris*: genes associated with cellular motility, sporulation, and ribosome function were downregulated (Song et al., 2019). This was different from the antibacterial action of nisin which targets membrane transporters, membrane, and cell wall synthesis and energy metabolism, suggesting a novel and improved antibacterial mechanism of the IONPs@pDA-Nisin. Another type of novel antibacterial ENM, pegylated silver-coated single-wall carbon nanotubes (PEG-SWCNT-Ag), was also tested against a foodborne pathogen, *Salmonella enterica* serovar Typhimurium, and proteomics proved useful for revealing not only the antibacterial mechanisms but also compensatory reactions in the surviving bacteria (Park et al., 2018). Specifically, the pathways that were activated to counteract the PEG-SWCNT-Ag effects included membrane rearrangement, elimination of ROS, and optimization of metabolic pathways toward acquiring energy and nutrients from alternative sources. These examples suggest that omics techniques can serve as useful guiding tools for the design of novel antibacterial ENMs.

The antibacterial action of soluble metal-based NPs, such as Ag, ZnO, and CuO NPs, has been attributed mainly to the release of metal ions (Bondarenko et al., 2013; Ivask et al., 2014) and several omics studies have consistently shown a relatively pronounced bacterial response. For example, in the case of ZnO NPs, mainly Zn^2+^-mediated antibacterial mechanisms were identified in a transcriptomic study of the cyanobacterium *Synechococcus elongatus* exposed to two types of ZnO NPs with different solubilities or ZnCl_2_ at growth-inhibitory concentrations (Vicente et al., 2019). Specifically, all Zn compounds disturbed the cell membrane potential, resulting in impaired photosynthesis and respiration, and the compounds also induced oxidative stress that led to lipid peroxidation and DNA damage, suggesting the role of Zn^2+^ as the main contributor to the ZnO NP toxicity. The main role of Zn^2+^ in the antibacterial action of ZnO NPs was also demonstrated in a proteomic study using a Gram-positive bacterium—*Bacillus subtilis* (Luche et al., 2016). Exposure to ZnO NPs or ZnSO_4_ at equitoxic concentrations which reduced the bacterial viability by 50% revealed that the proteome-level response in the surviving bacteria involved reorientation of central metabolism to protect the cell. Although the levels of thiols, which bound excessive Zn^2+^ in the cells, were upregulated, Zn compounds induced oxidative stress.

Commonly, the excessive metal ion concentrations in the cell are associated with the generation of intracellular ROS. Metal-based ENMs can act as sources for metal ions which, upon internalization, disturb the intracellular redox balance. This mechanism of action was proposed for Ag NPs in a proteomics study of a multidrug-resistant *P. aeruginosa* exposed to Ag NPs at twofold minimal inhibitory concentration (MIC) (Liao et al., 2019). The ROS-related antibacterial mechanism of Ag NPs was also detected in another proteomics study with *P. aeruginosa* exposed to Ag NPs or Ag^+^ at their respective EC_20_ based on viability at 24 h, where Ag^+^, differently from Ag NPs, did not elevate the levels of cellular ROS or oxidative stress proteins (Yan et al., 2018). The likely cause for the NP-specific ROS-mediated mechanism could have been the continuous release of Ag^+^ from the cell-attached NPs which resulted in sufficiently high levels of intracellular Ag^+^ to generate ROS. Other regulated pathways, mainly related to membrane proteins, were similar in Ag NP- and Ag^+^-exposed *P. aeruginosa*. The results suggested that the NP-specific antibacterial activity of Ag NPs was caused by the continuous and excessive release of ionic Ag.

In the cyanobacterium *Microcystis aeruginosa*, Ag NP exposure at 20% growth-inhibitory concentration caused mostly similar metabolic changes as Ag^+^ exposure, suggesting that released Ag^+^ contributed to the antimicrobial effects of Ag NPs (Zhang et al., 2018). However, there were differences in Ag NP- and Ag^+^-induced metabolite levels in indole alkaloid and steroid biosynthesis as well as phospholipid, arginine and proline metabolism which indicated adaptive responses of *M. aeruginosa* to oxidative damage and membrane stress exerted by Ag NPs. In another study of Ag NP-exposed cyanobacterium *M. aeruginosa*, proteomics analysis indicated that Ag NPs at a growth-inhibitory (60%) concentration downregulated the expression of proteins responsible for carbohydrate metabolism, translation, oxidative stress, membrane transport, and shikimate pathway which indicated that the antioxidant potential of the cyanobacteria was compromised (Qian et al., 2016). Thus, the response of the same bacterial strain—*M. aeruginosa*— to Ag NP-induced oxidative stress depended on the ENM effect level: ROS-detoxifying pathways were upregulated at 20% growth inhibition (Zhang et al., 2018) but suppressed at 60% growth inhibition (Qian et al., 2016). In both studies, Ag NP exposure significantly affected the photosynthesis process in *M. aeruginosa*, however, the underlying mechanisms were different: interference of porphyrin and chlorophyll metabolism at 20% growth inhibition (Zhang et al., 2018) and decreased activity of ROS-detoxifying enzymes at 60% growth inhibition (Qian et al., 2016). Ag NP-induced suppression of photosynthesis was also demonstrated using a transcriptomic analysis of a freshwater plankton community (Lu et al., 2020). Specifically, metabolic pathways related to those in cyanobacteria were significantly downregulated after 7-day exposure to Ag NPs at a concentration which reduced the content of chlorophyll *a* by ∼24%. Overexpression of proteins related to the biosynthesis of unsaturated fatty acids, branched-chain amino acids, vitamin B_6_, biotin and cobalamin in bacteria in the microcosm was attributed to detoxification mechanisms against Ag NP damage.

In addition to apparent similarities in the action of Ag NPs and Ag^+^, omics studies have also identified differences in the biological responses to the two Ag compounds. Proteome profiling of two different *Pseudomonas* strains exposed to Ag NPs or Ag^+^ at equitoxic growth-inhibitory concentrations indicated that Ag^+^ affected the expression of membrane transport proteins more strongly than Ag NPs (He et al., 2014; Barros et al., 2019). Additionally, He et al. (2014) demonstrated that exposure to Ag^+^ or graphene-attached Ag NPs similarly suppressed proteins involved in the translation process, whereas Barros et al. (2019) showed that Ag NPs upregulated, while Ag^+^ downregulated, the proteins associated with the translation process in *Pseudomonas* sp. Even though two different types of Ag NPs were studied, one of the factors contributing to the discrepancies between the results of the studies may have been the extent of Ag^+^ released from the specific Ag NPs used in these studies.

Omics analyses conducted at inhibitory ENM concentrations have also identified defense mechanisms that bacteria have activated in response to commonly employed soluble metal-based ENMs. Similar to upregulation of detoxification mechanisms in response to Ag NP exposure (Zhang et al., 2018; Lu et al., 2020), bacteria have upregulated cellular pathways in defense against CuO (Lu et al., 2013) and ZnO NPs (Luche et al., 2016). A number of virulence genes were upregulated by *Legionella pneumophila*, the causative agent of Legionnaires’ disease, upon exposure to CuO NPs at a growth-inhibitory concentration (Lu et al., 2013). Considering that increased virulence has been suggested to play a role in the pathogenesis of bacteria and help evade inactivation by phagocytes in mammalian hosts, CuO NPs may exacerbate *L. pneumophila* infections (Chang et al., 2007). In addition, several proteins involved in the stringent response, associated with the regulation of virulence and pathogenic processes, showed a modified expression profile in ZnO NP-exposed *B. subtilis*, suggesting that ZnO NPs can modulate these important bacterial functions (Luche et al., 2016).

For non-soluble metal-based ENMs, both ROS-dependent and -independent antibacterial mechanisms have been reported, depending on the exposure conditions. For example, in the case of typical photocatalytic ENMs, TiO_2_ NPs, omics investigations have confirmed the oxidative stress-based antibacterial mechanism under visible or ultraviolet light (Planchon et al., 2017). In addition, by using transcriptomic and proteomic approaches it was revealed that TiO_2_ NP exposure in the dark induced osmotic stress, changes in the metabolism of cell envelope components, and uptake/metabolism of endogenous and exogenous compounds in *E. coli* (Sohm et al., 2015). By strongly interacting with the bacterial cell membrane, TiO_2_ NPs induced depolarization and loss of membrane integrity which resulted in the leakage of K^+^ and Mg^2+^ and depletion of intracellular ATP, ultimately causing loss of bacterial viability. ROS-independent antibacterial mechanisms have been identified also in the case of Au NPs. Transcriptomics indicated that surfaces coated with nanoporous Au exerted antibacterial effects against *E. coli* by disruption of cell membranes via catalytic processes (Hakamada et al., 2017) and a combination of transcriptomics and proteomics analyses indicated that Au NPs were antibacterial against *E. coli* through changing the membrane potential and inhibiting ATP synthase (Cui et al., 2012). An ROS-independent mechanism of action was considered to be responsible for the low toxicity of Au NPs to mammalian cells, and thus would allow the development of antibacterial agents with low toxic side effects to non-target eukaryotic cells. A similar approach has already been demonstrated *in vivo* with quaternary ammonium carbon quantum dots (QCQDs) which were effective specifically against Gram-positive bacteria (methicillin-resistant *Staphylococcus aureus* or MRSA) without inducing excessive ROS and showed good biocompatibility in mice (Zhao et al., 2020). Proteomics enabled identifying the interference with bacterial ribosomes and RNA degradation as the antibacterial mechanism of the novel QCQDs which showed low cytotoxicity toward eukaryotic cells due to their different ribosome structures. These studies demonstrated how non-targeted analysis of ENM antibacterial mechanisms can be useful for the design of a novel antibacterial ENM.

ENM antibacterial mechanisms have been extensively studied in oxic conditions which are relevant for topical applications in fighting pathogens or aquatic ecosystems receiving ENM pollution. However, due to different ENM physico-chemical transformations and bacterial physiology the antibacterial mechanisms may differ in anoxic conditions. A few studies have employed omics methods to explore ENM mechanisms in anoxic environments, for example in the denitrification process (Zheng et al., 2018). Exposure of *Paracoccus denitrificans* to Ag NPs at growth-inhibitory levels resulted in downregulation of genes and proteins related to denitrification, glycolysis, and electron transfer, indicating that energy metabolism was suppressed (Zheng et al., 2018). Increased intracellular polyhydroxybutyrate (PHB) synthesis contributed to impaired denitrification because of competition for carbon sources. Although oxidative dissolution of metallic Ag is expected to be negligible at low or depleted oxygen levels, Ag NPs were shown to release Ag^+^ in anoxic conditions in the medium containing nitrate and nitrite which can act as terminal electron acceptors. This suggests that Ag^+^ could have contributed to the observed effects of Ag NPs to bacterial denitrification. On the contrary, Cu^2+^ released from CuO NPs in anoxic conditions did not have a significant inhibitory effect on the growth or denitrification activity of *P. denitrificans* (Su et al., 2015). Proteomics analysis revealed that the underlying mechanisms of CuO NPs in inhibiting denitrification included downregulation of proteins associated with nitrogen metabolism, electron transfer, and transport.

#### Omics Studies With Bacteria at Sub-Inhibitory ENM Effect Levels

Employing omics assays with bacteria that have been exposed to ENMs at concentrations which induce no growth inhibition, membrane damage, or loss of viability, has a potential to discover subtle changes in cell physiology and regulation induced by ENMs (Supplementary Table 1). The results can provide information about the consequences of low-concentration, chronic exposures and also help to discover new biomarkers for ENM toxicity. These studies are usually conducted on beneficial bacteria to elucidate potential effects of ENMs at low level exposures resulting either from incidental release or intentional application, e.g., in agriculture.

Omics techniques have enabled identifying differences in the transcriptional level responses of bacteria to exposures of ENMs at different sub-inhibitory exposure concentrations (Mortimer et al., 2020). In addition, concentration-dependent regulation of the transcriptome was shown to vary for different ENMs, depending on their physico-chemical properties and extent of agglomeration (Wang et al., 2018b) which may reduce ENM bioavailability (Wang et al., 2017) and consequently biological responses in bacteria (Mortimer et al., 2020). However, ENMs are also known to form heteroagglomerates with bacteria which can increase bacterial-ENM interactions. For example, MWCNTs at a sub-growth-inhibitory concentration were shown to induce pronounced transcriptomic responses in *P. aeruginosa* via interactions in MWCNT-bacterial heteroagglomerates (Mortimer et al., 2018). Changes in gene expression indicated reduced motility and membrane transport effects in *P. aeruginosa* as well as upregulation of sulfur metabolism and downregulation of an outer membrane porin, indicative of anoxic conditions, possibly caused by ENM-bacterial heteroagglomeration. The finding that MWCNTs downregulated the gene encoding the outer membrane porin associated with carbapenem and heavy metal resistance enabled establishing a connection between MWCNT exposure and increased antibiotic susceptibility of *P. aeruginosa*, confirmed by exposing bacteria to MWCNTs and antibiotics. This study thus demonstrated that transcriptomics can serve as a sensitive tool in revealing ENM effects that could be useful in fighting antibiotic resistance. On the other hand, there are reports on antagonistic activity of oxidized MWCNTs and organic compounds such as the pesticide pentachlorophenol (Deng et al., 2019) and the antibiotic ciprofloxacin (Deng et al., 2020). Transcriptomics and a combination of transcriptomics and metabolomics, respectively, indicated that MWCNTs attenuated the pentachlorophenol-induced disturbances in gene expression (Deng et al., 2019) and offset the impact of ciprofloxacin in *E. coli* gene expression and its metabolome (Deng et al., 2020). The different outcomes of MWCNT exposures to the toxicity of organic compounds in bacteria may be caused by the physico-chemical properties of these compounds that influence their interactions with MWCNTs and bacteria, or depend on whether pre- or co-exposures of MWCNTs with the antibiotics were conducted. Overall, the combined effects of ENMs and other toxicants to bacteria may be complex, and omics techniques appear to be excellent tools for shedding light on this environmentally and ecologically important topic.

Another area where omics techniques have proved essential is exploring the associations between symbiotic bacteria and their hosts. At low, environmentally relevant concentrations, ENMs may not affect the growth or viability of environmental bacteria but may interfere with host-bacteria signaling, such as characteristic to the plant growth promoting rhizobia (PGPR). For example, ENM type- and concentration-dependent regulation of gene expression levels was detected in the soybean symbiotic N_2_-fixing bacterium *Bradyrhizobium diazoefficiens* by using transcriptomic analysis (Mortimer et al., 2020). The gene expression level changes indicated that MWCNTs and rod-shaped CeO_2_ NPs, two ENMs with different chemical composition but similar tubular shape and agglomeration state, significantly altered the root nodulation competitiveness of *B. diazoefficiens*. Spherical carbon black NPs and planar graphene nanoplatelets, tested in the same study, on the other hand, were less potent in inducing transcriptomic changes, suggesting that the tubular shape of MWCNTs and CeO_2_ NPs facilitated better cell-ENM contact to hinder metabolic fitness and energy metabolism of the bacteria. Another PGPR—the soil bacterium *B. subtilis*—was shown to be affected by Ag NPs at sub-inhibitory concentrations based on a proteomics assay (Gambino et al., 2015). Ag NPs, added to *B. subtilis* biofilm, stimulated the expression of proteins associated with the stress response or sensing of cellular redox potential and promotion of quorum sensing. Upregulation of the structural components of several proteins such as alkyl hydroperoxide reductase, Fe-S clusters, and thioredoxin suggested that Ag NPs induced an adaptive response for efficient detoxification of ROS. The results suggested that Ag NPs at sub-inhibitory concentrations can affect essential cellular processes in the rhizosphere. Adaptive responses to ENM exposure at sub-inhibitory levels in *B. subtilis* biofilm were also reported in the case of Mg doped ZnO (Mg-ZnO) NPs (Auger et al., 2019) and Al_2_O_3_ NPs (Mu et al., 2016). Proteomic analysis indicated that Mg-ZnO NPs induced the expression levels of proteins associated with ROS detoxification, translation, and biofilm formation, while transcriptomics of Al_2_O_3_ NP-exposed *B. subtilis* biofilms suggested induction of DNA repair, the ROS scavenger system, and flagellar biosynthesis, as well as surfactin synthesis and biofilm formation, to adapt to or evade ENM-induced stress.

The most often studied ENM type at sub-inhibitory effect levels was Ag, similarly to the studies using inhibitory ENM levels. A novel gel- and label-free proteomic approach was used to explore the effects of sublethal concentrations of Ag NPs on *E. coli* biofilms as a model for gut bacteria (Domingo et al., 2019). The new method yielded 212 differentially expressed proteins which is approximately a 6 times higher value than the average number of differentially expressed proteins in all the other proteomics studies using sub-inhibitory concentrations of ENMs, suggesting that advanced technologies may benefit omics approaches with increased detection sensitivity. The experimental setup of the study (acute: 1 mg/L for 24 h, chronic: 1 mg/L for 96 h, and chronic + acute: 1 mg/L for 72 h + 10 mg/L for 24 h, exposures to Ag NPs) allowed for studying the time-dependent evolution of protein up- and downregulation which is a different approach from most omics studies usually conducted at one time point. The main biological processes found to be affected by Ag NPs in the *E. coli* biofilm included glucose metabolism, DNA repair, stress response, cell wall and biofilm structure. Sublethal concentrations of Ag NPs appeared to compromise biofilm formation by downregulating flagellins and fimbrial proteins which are responsible for bacterial adhesion. In addition to *E. coli* biofilms, sub-inhibitory concentrations of Ag NPs have been shown to exert harmful effects to *E. coli* planktonic cells at the gene expression level. Ag NP effects on *E. coli* were compared to these of Ag^+^ and, based on the transcriptomic profiles, the mechanism of Ag NPs was attributed to Ag^+^ dissolution at the cell wall resulting in enhanced interfacial silver concentration that entered the cell (McQuillan and Shaw, 2014). Intracellularly, Ag^+^ caused protein unfolding and induced redox stress as inferred from the upregulation of genes encoding heat shock response and proteins containing Fe-S clusters. Ag NP-associated mechanisms were different from AgNO_3_-induced effects, suggested to be caused by a different mode of Ag^+^ delivery into bacteria for each silver compound. Other harmful effects that have been associated with bacterial exposures to Ag^+^ or Ag NPs at sub-inhibitory concentrations include changes in the frequency of the horizontal transfer of antibiotic resistant genes (ARGs) as was recently reported for the donor-recipient pair of *E. coli* and *P. putida* (Lu et al., 2019). Transcriptomics and proteomics analyses revealed that Ag NPs and Ag^+^ promoted horizontal gene transfer by generating elevated levels of intracellular ROS, cell membrane permeability, and the SOS response. These results alert to the potential ecological risks of ENMs in increasing the dissemination of ARGs, especially given that other types of ENMs besides Ag NPs have been shown to induce oxidative stress and membrane damage in bacteria.

An ROS-dependent antibacterial mechanism is especially prevalent in the case of photoactive ENMs; however, other mechanisms may also play a role. For example, a transcriptomics approach was used to determine the specific contributions of oxidative stress and NPs in the antibacterial mechanism of phototherapeutic CdTe QDs (Aunins et al., 2019). The gene expression profiles of *E. coli* exposed to CdTe QDs under light or dark conditions or to non-photoactive CdSe QDs were compared, indicating that bacteria responded to superoxide generation from illuminated CdTe QDs by transcriptional changes associated with DNA repair and deactivation of enzymes with Fe-S clusters. In contrast, the response observed exclusively upon CdSe QD exposure included downregulation of leucyl-tRNAs which was interpreted as part of a stress response in *E. coli* to slow the translation process with the aim to prevent protein misfolding or aggregation, or reduce growth to conserve resources (Aunins et al., 2019). Interestingly, downregulation of several tRNA genes, including those of leucyl-tRNAs, was also found in the transcriptomics analysis of MWCNT- and GNP-exposed symbiotic N_2_-fixing bacteria *B. diazoefficiens* (Mortimer et al., 2020). Similarly to the case of CdSe QDs, suppression of tRNA gene transcription may have been a general stress response of bacteria to MWCNTs and GNPs to optimize energy consumption, but there was also a possibility that the ENMs interfered with plant–microbe signaling and nodulation efficiency since it has been reported that tRNA fragments function as nodulation signal molecules in *B. diazoefficiens* (Ren et al., 2019a). This implies that there is a large degree of uncertainty in interpreting transcriptional changes, including in response to ENMs, since much of the functional information remains scarce.

Certain bacterial strains have higher tolerance to metals and to metal ENMs (Priester et al., 2009). Omics approaches have enabled understanding the resistance or adaptation mechanisms to ENMs in bacteria. For example, proteomics analysis revealed the mechanisms of coping with CdSe NP stress in a marine bacterium *P. fluorescens* (Poirier et al., 2016). Cd^2+^ release from the CdSe QDs was evidenced by the upregulation of cadmium-exporting ATPase, even though analytical measurements did not indicate metal ion release from the QDs in the presence of bacteria. The reason was likely the efficient sequestration of CdSe QDs and ions in the exopolysaccharides and surface glycostructures which were upregulated. Still, it was concluded that Cd^2+^ and Se^2–^ accumulated in the cell, because the soxRS regulatory system, luciferase, metallothioneins, glutathione, catalase hydroperoxidase I, and selenoproteins were all upregulated which suggests a defense mechanism against oxidative stress, usually induced by excess of metal ions. Meanwhile, several transmembrane transporters and iron acquisition systems were downregulated to inhibit CdSe QD uptake in the cells, and the synthesis of lipopolysaccharides and phospholipids was reduced to hinder QD adsorption on the cell surface. Owing to the effective adaptation mechanisms and the ability to sequester relatively large amounts of QDs, *P. fluorescens* was proposed as a promising bioremediation agent. However, in natural environments, accumulation of CdSe QDs or other types of ENMs in or on bacteria can pose a threat of bioaccumulation or trophic transfer of ENMs which can have ecological impacts (Priester et al., 2009; Werlin et al., 2011; Mortimer et al., 2016).

Similar proteomic responses as with CdSe QD-exposed *P. fluorescens* were detected in a lactic acid bacterium *Lactobacillus reuteri* incubated with Se NPs at sub-inhibitory concentrations (Gomez-Gomez et al., 2019). The similarity was mainly in upregulation of thioredoxin to protect the cell from excessive ROS, as well as increased synthesis of exopolysaccharides known to bind selenium. These changes together with upregulation of proteins related to selenium metabolism, upon exposure of *L. reuteri* to either Se NPs or selenite, indicated that Se NPs exerted its effects, at least partly, through dissolved selenium ions. Further, selenite was shown to have more deleterious effects than Se NPs on this lactic acid bacterium.

Since ROS-mediated antibacterial action of metal ENMs is realized through released metal ions that enter cells, oxidative stress induction by ENMs is not likely in anaerobic environments where ENM dissolution is limited. This was demonstrated to be the case in CuO NP-exposed *Desulfovibrio vulgaris*, a model sulfate-reducing bacteria, where transcriptomic assays did not indicate any changes that would have been induced in oxidative stress conditions (Chen et al., 2019). The bacterium showed high tolerance to CuO NPs, suggesting that the strain could have a potential application in bioremediation.

#### Omics Studies With Bacteria at Stimulatory ENM Effect Levels

Omics approaches can also help with understanding mechanistic aspects of ENM stimulatory properties which can be used to enhance beneficial functions of bacteria. Such beneficial effects of ENMs have been reported in enzyme production, wastewater treatment, and environmental remediation. Surprisingly, Ag NPs which typically exert antibacterial effects, were found to have a stimulatory effect on the anaerobic ammonium oxidizing (anammox) process in bacterial granules (Peng et al., 2019). Ag NPs acted by increasing the porosity of the anammox granules which enhanced the diffusion of nutrients and iron ions into the granules which, in turn, upregulated anammox-related enzyme expression as identified using proteomics analysis. The formation of pores or cavities in the anammox granules was caused by the selective bactericidal activity of Ag NPs toward certain bacterial taxa and relative resistance of anammox bacteria to Ag NPs due to the ability to synthesize encapsulins which can bind Ag^+^ and reduce toxicity. Upregulation of anammox-related enzymes contributed to the enhanced metabolism and nitrogen removal performance while bacterial population growth increased. Based on this mechanism, the anammox process was proposed as a promising method for the treatment of Ag NP-containing wastewater. Another ENM which is commonly used as an antibacterial agent, CuO, was also found potentially applicable in the biotreatment of wastewater. Specifically, CuO NPs exerted stimulating effects on the Cr(VI) reduction process by *Cupriavidus basilensis* B-8 (Yan et al., 2019). The strain had high tolerance for Cr(VI) which was further enhanced in the presence of CuO NPs. Transcriptomics analysis revealed the underlying mechanism for such an effect: CuO NPs induced additional stress which caused the bacteria to adapt by upregulating the genes encoding sulfur- or nitrogen-containing proteins that play an important role in Cr(VI) reduction, genes related to Cr(VI) resistance and genes encoding redox enzymes. Most of the upregulated proteins were involved in either specific or non-specific reduction of Cr(VI). Further, the stimulatory effect was NP-specific since Cu^2+^ did not induce a similar induction in Cr(VI) reduction capacity of *C. basilensis* B-8.

ENMs have also been implicated in stimulating polycyclic aromatic hydrocarbon (PAH) degradation by bacteria. Graphene oxide QDs (GOQDs) which are composed of aromatic hydrocarbon structures were used to selectively isolate a PAH-degrading bacterial strain *Bacillus cereus* from a contaminated soil (Mu et al., 2019). Subsequent incubation of the bacterial strain with GOQDs stimulated bacterial proliferation and accelerated PAH degradation. Based on proteomic analysis, GOQDs induced the overexpression of microbial divisomal proteins associated with division initiation, DNA replication and peptidoglycan hydrolysis or synthesis. Since the GOQD-induced effects lasted for 20 generations without the addition of GOQDs, it appears that the isolated strain was especially efficient in PAH degradation due to exposure to GOQDs in the initial strain selection process, or that the intracellular GOQDs that had entered the bacterial cells during the initial exposure were transferred to the next generations and continued to exert selective pressure without the need for added GOQDs. The third explanation for the effects lasting over 20 generations could be that the GOQD-induced changes involved genetic mutations that were passed on to the next generations. Although a mutation analysis of the GOQD-exposed *B. cereus* was not conducted (Mu et al., 2019), ENM-induced genetic mutations in bacteria have been reported. For example, the changed transcript sequence was one of the underlying mechanisms of the stimulatory effect of Mg–Fe layered double hydroxide (Mg–Fe LDH) in the marine bacterium *Arthrobacter oxidans* which increased its exodextranase yields upon ENM exposure (Ren et al., 2019b). Namely, transcriptomics revealed that exposure to Mg–Fe LDH led to formation of a new Shine-Dalgarno sequence (ribosomal binding site in bacterial messenger RNA) that influenced the expression of dextranase. Mg–Fe LDH effectively adsorbed on the bacterial surface and released Fe^3+^ which affected the expression of transcripts related to ferric iron metabolism; genes associated with siderophore synthetase, iron complex transport system and Fe-S cluster assembly were all downregulated. Since LDHs have a high specific surface area, they have been explored as potential agents for removing environmental contaminants. However, the transcriptomics study with LDH-exposed *A. oxidans* identified the previously undiscovered effect of LDHs—enhancement of the dextranase production by a marine bacterium—which suggests a whole new area of application for this type of ENM.

#### Comparison of Bacterial Pathway-Level Responses to ENMs at Inhibitory, Sub-Inhibitory, and Stimulatory Effect Levels

Based on the studies reviewed above, oxidative stress associated cellular processes as measured by omics approaches were affected by ENMs in many studies conducted either at inhibitory, sub-inhibitory or stimulatory exposure levels which corroborates the often-proposed oxidative stress-mediated antibacterial mechanism of ENMs. However, there were some apparent differences in the cellular and molecular targets of oxidative stress-inducing ENMs in the studies conducted at inhibitory vs. sub-inhibitory or stimulatory ENM exposure concentrations. For example, one of the apparent differences which emerged when reviewing the published literature was the aspect that the studies conducted at sub-inhibitory and stimulatory, but not inhibitory, concentrations of metal ENMs, reported dysregulation of Fe-S clusters which are protein cofactors that act as redox sensors. Fe-S clusters have been reported to be sensitive to ROS and metal ions such as copper, silver, cadmium and zinc (Xu and Imlay, 2012; Vernis et al., 2017), which explains the dysregulation of these proteins in several studies with Ag NPs (McQuillan and Shaw, 2014; Gambino et al., 2015) and a study involving CdTe QDs (Aunins et al., 2019). Also, Mg-Fe LDHs at stimulatory concentrations were reported to downregulate the Fe-S cluster assembly (Ren et al., 2019b). The primary role of Fe-S clusters is electron transfer, but also transcriptional and translational regulation via sensing the changes in cellular redox conditions (Kiley and Beinert, 2003). The apparent regulation of these metalloproteins at sub-inhibitory and stimulatory rather than inhibitory ENM exposure conditions may be related to the differences in posttranscriptional regulation in bacteria at the different ENM effects. Also, KEGG pathways associated with transcription and translation were more often affected at sub-inhibitory than inhibitory ENM concentrations as was revealed by the comparative analysis of the pathway-level effects of ENMs discussed below. Thus, the observation of Fe-S cluster regulation at lower rather than higher ENM concentrations illustrates a general trend in the differences between the ENM effective categories.

**FIGURE 3:**
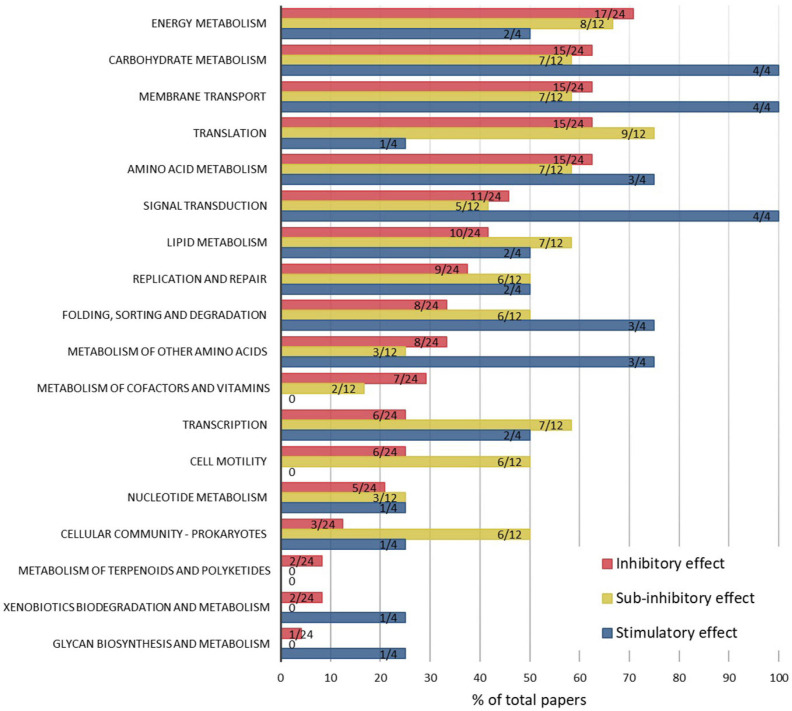
Percentages of papers which report ENM-induced pathway modulations, by KEGG pathways and ENM effect levels, which were associated with inhibitory (24 papers), sub-inhibitory (12 papers), or stimulatory (4 papers) outcomes to the physiology (mainly growth or viability) of bacteria. The data labels indicate the number of papers reporting the affected pathway out of the total number of papers in each of the three categories. Raw data are summarized in Supplementary Table 2.

To compare the pathway-level effects of ENMs associated with inhibitory, sub-inhibitory, and stimulatory effects, the results from all omics studies across different ENM types, bacterial strains and exposure conditions were compiled (Figure 3). Despite different numbers of publications in the “inhibitory,” “sub-inhibitory,” and “stimulatory” effects categories, and thus different representativeness of the affected KEGG pathways in each category, the comparison gives an overview of general trends in ENM effect-dependent regulation of cellular processes. In both inhibitory and sub-inhibitory ENM effect categories, the majority of the published studies (∼60%) reported changes in energy and carbohydrate metabolism, membrane transport, translation and amino acid metabolism, reflecting ENM effect-independent regulation of major cellular processes. However, many of the other KEGG pathways were affected more often at sub-inhibitory than inhibitory ENM effect levels: these pathways included lipid metabolism, replication, and repair, folding, sorting and degradation, transcription, cell motility and prokaryotic cellular community. ENMs at stimulatory effect levels were used only in four studies, but all studies indicated changes in carbohydrate metabolism, membrane transport and signal transduction, obviously important in inducing beneficial changes in bacteria. Three studies out of four in the “stimulatory” category also reported the regulation of amino acid metabolism and sorting and degradation, suggesting the importance of posttranscriptional processes to stimulatory conditions.

Cellular responses in bacteria at sub-inhibitory ENM effect levels appear to include a wider range of metabolic pathways than at inhibitory ENM levels, based on the number of studies reporting each pathway (Figure 3). This might be attributed to the aspect that in toxic conditions bacterial energy consumption is directed to regulating central metabolism crucial for cellular survival, whereas at sub-inhibitory conditions a range of pathway changes are activated to adapt to ENMs and to achieve a new steady-state in the synthesis and degradation of proteins and metabolites. Remarkably, cell motility and cellular community functions, which include quorum sensing and biofilm formation, were affected in 50% of the studies conducted at sub-inhibitory conditions but only in 10–20% of studies where ENMs were used at inhibitory levels. Since biofilm is a dominant life form of bacteria, it is important to understand the regulatory mechanisms involved in, and physiological implications resulting from, ENM exposures at sub-growth-inhibitory or sublethal levels. For example, low concentrations of ZnO NPs were shown to stimulate biofilm formation by upregulating quorum sensing genes, among others (Ouyang et al., 2020), which may have implications such as unintended promotion of bacterial biofilms. Also, a proteomics study revealed suppressive effects of CdSe NPs at sub-inhibitory concentrations on pyoverdine secretion in a marine bacterial strain of *P. fluorescence* (Poirier et al., 2016). Pyoverdine is a siderophore with a key role in bacterial survival and competitiveness in ecosystems and also a virulence factor in pathogenic strains such as *P. aeruginosa*, so its regulation has both environmental and clinical implications. Thus, omics approaches, especially when used at sub-inhibitory ENM effect levels, can provide crucial information on ENM effects to bacterial secondary metabolism and the potential environmental and human health outcomes. Overall, the comparison of the affected KEGG pathways at different “ENM effect” categories indicated that the mechanisms of action of ENMs in bacteria depend on ENM exposure concentrations which differently affect the growth or viability of bacteria.

## Discussion

### Challenges and Opportunities of Omics Methods

As demonstrated in this review of omics analyses of ENM-exposed bacteria, most studies so far have assessed ENM effects at a single time point and concentration, limiting the temporal and dose-dependent mechanistic understanding gained from omics measurements. Conducting the analyses at several concentrations and time points may also help to shed light on antibacterial mechanisms vs. bacterial defense mechanisms to ENM exposures. Commonly, bacterial cultures subjected to omics analyses are heterogeneous in terms of physiological state, especially when exposed to ENMs at inhibitory concentrations, because ENMs interact with bacterial populations unevenly thus causing death or damage of some bacteria while others survive and develop stress responses. As a result, omics data consist of pooled molecular responses to ENM exposure which may not occur in the same fraction (dying or surviving) of the bacterial population, making the results difficult to interpret. Such a situation was described in the case of a TiO_2_ NP-exposed *E. coli* culture where part of the bacterial population was shown to strongly interact with NPs while the other part was totally deprived of NPs on the cell surface (Planchon et al., 2017). Thus, the discrepancies in the detected changes in protein expression and metabolite levels were attributed to the heterogeneity of the culture, and the results were interpreted as corresponding to a collective response of the population where some bacteria were sacrificed for the rest to survive the exposure to TiO_2_ NPs. To address the issue of population heterogeneity in ENM-exposed bacterial cultures, especially if inhibitory concentrations are used, future studies could include a separation step to sort the dead or damaged cell and live cell populations, for example, using differential staining and flow cytometry, before conducting omics assays. This approach would allow for identifying and isolating acute molecular responses in damaged cells separately from the adaptation strategies in the surviving cells.

Advances in connecting cellular functions with transcriptional, proteomic and metabolomic changes in bacteria have enabled the application of these technologies in nanotoxicology. However, the detailed molecular mechanisms and signaling cascades of many cellular pathways are still unknown which causes some uncertainty in interpretation of omics data sets. As new discoveries are made on the functions of genes and roles of proteins and metabolites, as well as regulatory roles of non-coding RNA molecules, e.g., small RNA or sRNA (Ryan et al., 2020) and fragments of tRNA (Ren et al., 2019a), conducting meta-analyses of already obtained omics data could serve as a means of gaining additional and more accurate functional information on affected pathways in ENM-exposed bacteria. Such an approach has already been taken with transcriptomic data of eukaryotic model organisms that had been exposed to ENMs (Burkard et al., 2020). Remarkably, out of 46 environmentally relevant transcriptomic studies with publicly available data studied by Burkard et al. (2020), only 11 complete microarray data sets were considered suitable for reanalysis (i.e., had a minimum of three replicates, available genomic annotation, complete technical information, and raw data). Thus, to help bridge the gap between the currently unknown functional pathways and signaling cascades and future discoveries, it is crucial to make the existing data available and accessible for meta-analysis, as well as to scrutinize the experimental protocols in terms of including proper controls and sufficient number of replicates.

Comparison across published studies is further complicated by different statistical criteria applied in identifying the significant changes in gene transcription, protein expression, or metabolite levels. For example, some studies use *p*-values based on *t*-tests while others perform analysis of variance (ANOVA) with a set false discovery rate (FDR). The criteria used for identifying differentially expressed genes or proteins may thus influence the outcome of the study and either over- or underestimate the extent of changes. Setting a unified protocol and parameters for the statistical analysis of omics datasets would facilitate the comparison of the results of different studies. Additionally, the results may be compromised by the technical limitations in omics methodologies; for example, caused by low sensitivity when determining changes in protein expression levels based on quantification of band intensities in the gel (Dowsey et al., 2010). However, older analytical methods are being replaced with techniques that have improved sensitivity, throughput, and accuracy (such as replacement of microarrays with RNAseq in transcriptomics or 2D gel-based protein quantification with shotgun methods using LC-MS or data-independent acquisition methods like SWATH-MS for quantitative proteomics) while more advanced statistical analysis methods are also implemented (Costa-Silva et al., 2017; Wen et al., 2017; Mock et al., 2018; Zhu et al., 2020).

### Concluding Remarks

Omics methods have revealed molecular level changes in bacteria upon ENM exposure that would have not been detected using other methods, for example regulation of important intra- and interspecies signaling molecules in bacteria, increased susceptibility to antibiotics or affected competitiveness in establishing plant-bacterial symbioses. Also, omics analyses have enabled identifying novel antibacterial mechanisms of ENM-enabled antibacterial compounds. Even though the test conditions, bacterial strains and ENM types differ across studies, comparison of the KEGG pathway-level changes induced by ENMs at three “effect” categories, i.e., inhibitory, sub-inhibitory, and stimulatory, clearly demonstrated the concentration-dependent trends in the regulated pathways. While growth-inhibitory or lethal concentrations of ENMs mainly affected central processes such as energy and carbohydrate metabolism, membrane structure and transport, translation and amino acid metabolism, sub-inhibitory and stimulatory ENM levels additionally regulated signal transduction, transcription and folding, sorting and degradation. Interestingly, two pathways that emerged as often regulated at sub-inhibitory ENM concentrations were motility and cellular community which involves quorum sensing and biofilm formation. These findings indicate that ENM exposure concentrations and ENM effects on growth or viability clearly influence the mechanism of action of ENMs in bacteria. To gain a better understanding of how ENM effects may change over time and at different doses, future test designs should include wider coverage of these test parameters and also consider the aspect of heterogeneity of the bacterial population upon ENM exposures. Omics approaches have a potential to greatly advance the mechanistic understanding of bacterial-ENM interactions, which is needed, especially in light of the development of nanomedicine and the recently recognized importance of the microbiome in human health (Zhang et al., 2020), and an increasing need for sustainable agriculture where plant-symbiotic microorganisms and nano-enabled agrochemicals play important roles (Holden et al., 2018).

## Author Contributions

MM drafted the manuscript with contributions from YW and PH. All authors revised the manuscript and approved the manuscript for the publication.

## Conflict of Interest

The authors declare that the research was conducted in the absence of any commercial or financial relationships that could be construed as a potential conflict of interest.

## Footnotes

1 https://www.genome.jp/kegg/

## References

[B1] Auger S. Henry C. Pechaux C. Lejal N. Zanet V. Nikolic M. V. (2019). Exploring the impact of Mg-doped ZnO nanoparticles on a model soil microorganism *Bacillus subtilis*. *Ecotoxicol. Environ. Saf.* 182:109421. 10.1016/j.ecoenv.2019.109421 31301592

[B2] Aunins T. R. Eller K. A. Courtney C. M. Levy M. Goodman S. M. Nagpal P. (2019). Isolating the *Escherichia coli* transcriptomic response to superoxide generation from cadmium chalcogenide quantum dots. *ACS Biomater. Sci. Eng.* 5 4206–4218. 10.1021/acsbiomaterials.9b01087 33417778

[B3] Barros D. Pradhan A. Mendes V. M. Manadas B. Santos P. M. Pascoal C. (2019). Proteomics and antioxidant enzymes reveal different mechanisms of toxicity induced by ionic and nanoparticulate silver in bacteria. *Environ. Sci. Nano* 6 1207–1218. 10.1039/c8en01067f

[B4] Bondarenko O. Juganson K. Ivask A. Kasemets K. Mortimer M. Kahru A. (2013). Toxicity of Ag, CuO and ZnO nanoparticles to selected environmentally relevant test organisms and mammalian cells in vitro: a critical review. *Arch. Toxicol.* 87 1181–1200. 10.1007/s00204-013-1079-4 23728526 PMC3677982

[B5] Burkard M. Betz A. Schirmer K. Zupanic A. (2020). Common gene expression patterns in environmental model organisms exposed to engineered nanomaterials: A meta-analysis. *Environ. Sci. Technol.* 54 335–344. 10.1021/acs.est.9b05170 31752483 PMC6950232

[B6] Chang M. W. Toghrol F. Bentley W. E. (2007). Toxicogenomic response to chlorination includes induction of major virulence genes in *Staphylococcus aureus*. *Environ. Sci. Technol.* 41 7570–7575. 10.1021/es070929k 18044543

[B7] Cheeseman S. Christofferson A. J. Kariuki R. Cozzolino D. Daeneke T. Crawford R. J. (2020). Antimicrobial metal nanomaterials: From passive to stimuli-activated applications. *Adv. Sci.* 7:1902913. 10.1002/advs.201902913 32440470 PMC7237851

[B8] Chen Z. Y. Gao S. H. Jin M. Sun S. J. Lu J. Yang P. (2019). Physiological and transcriptomic analyses reveal CuO nanoparticle inhibition of anabolic and catabolic activities of sulfate-reducing bacterium. *Environ. Int.* 125 65–74. 10.1016/j.envint.2019.01.058 30710801

[B9] Costa P. M. Fadeel B. (2016). Emerging systems biology approaches in nanotoxicology: Towards a mechanism-based understanding of nanomaterial hazard and risk. *Toxicol. Appl. Pharmacol.* 299 101–111. 10.1016/j.taap.2015.12.014 26721310

[B10] Costa-Silva J. Domingues D. Lopes F. M. (2017). RNA-Seq differential expression analysis: An extended review and a software tool. *PLoS One* 12:e0190152. 10.1371/journal.pone.0190152 29267363 PMC5739479

[B11] Cui Y. Zhao Y. Y. Tian Y. Zhang W. Lu X. Y. Jiang X. Y. (2012). The molecular mechanism of action of bactericidal gold nanoparticles on *Escherichia coli*. *Biomaterials* 33 2327–2333. 10.1016/j.biomaterials.2011.11.057 22182745

[B12] Deng R. Gao X. Hou J. Lin D. (2020). Multi-omics analyses reveal molecular mechanisms for the antagonistic toxicity of carbon nanotubes and ciprofloxacin to *Escherichia coli*. *Sci. Total Environ.* 726:138288. 10.1016/j.scitotenv.2020.138288 32305750

[B13] Deng R. Zhu Y. Hou J. White J. C. Gardea-Torresdey J. L. Lin D. (2019). Antagonistic toxicity of carbon nanotubes and pentachlorophenol to *Escherichia coli*: Physiological and transcriptional responses. *Carbon* 145 658–667. 10.1016/j.carbon.2019.01.077

[B14] Domingo G. Villa F. Vannini C. Garuglieri E. Onelli E. Bracale M. (2019). Label-free proteomic approach to study the non-lethal effects of silver nanoparticles on a gut bacterium. *Front. Microbiol.* 10:2709. 10.3389/fmicb.2019.02709 31866956 PMC6906586

[B15] Dowsey A. W. Morris J. S. Gutstein H. B. Yang G.-Z. (2010). Informatics and statistics for analyzing 2-D gel electrophoresis images. *Methods Mol. Biol.* 604 239–255. 10.1007/978-1-60761-444-9_16 20013375 PMC3711025

[B16] Fadeel B. Farcal L. Hardy B. Vazquez-Campos S. Hristozov D. Marcomini A. (2018). Advanced tools for the safety assessment of nanomaterials. *Nat. Nanotechnol.* 13 537–543. 10.1038/s41565-018-0185-0 29980781

[B17] Fröhlich E. (2017). Role of omics techniques in the toxicity testing of nanoparticles. *J. Nanobiotechnol.* 15 84–84. 10.1186/s12951-017-0320-3 29157261 PMC5697164

[B18] Gambino M. Marzano V. Villa F. Vitali A. Vannini C. Landini P. (2015). Effects of sublethal doses of silver nanoparticles on *Bacillus subtilis* planktonic and sessile cells. *J. Appl. Microbiol.* 118 1103–1115. 10.1111/jam.12779 25702880

[B19] Gomez-Gomez B. Perez-Corona T. Mozzi F. Pescuma M. Madrid Y. (2019). Silac-based quantitative proteomic analysis of *Lactobacillus reuteri* CRL 1101 response to the presence of selenite and selenium nanoparticles. *J. Proteomics* 195 53–65. 10.1016/j.jprot.2018.12.025 30593931

[B20] Hakamada M. Taniguchi S. Mabuchi M. (2017). Antibacterial activity of nanoporous gold against *Escherichia coli* and *Staphylococcus epidermidis*. *J. Mater. Res.* 32 1787–1795. 10.1557/jmr.2017.157

[B21] He T. Liu H. Zhou Y. Yang J. Cheng X. Shi H. (2014). Antibacterial effect and proteomic analysis of graphene-based silver nanoparticles on a pathogenic bacterium *Pseudomonas aeruginosa*. *BioMetals* 27 673–682. 10.1007/s10534-014-9756-1 24961696

[B22] Hochella M. F. Mogk D. W. Ranville J. Allen I. C. Luther G. W. Marr L. C. (2019). Natural, incidental, and engineered nanomaterials and their impacts on the Earth system. *Science* 363:eaau8299. 10.1126/science.aau8299 30923195

[B23] Holden P. A. Gardea-Torresdey J. L. Klaessig F. Turco R. F. Mortimer M. Hund-Rinke K. (2016). Considerations of environmentally relevant test conditions for improved evaluation of ecological hazards of engineered nanomaterials. *Environ. Sci. Technol.* 50 6124–6145. 10.1021/acs.est.6b00608 27177237 PMC4967154

[B24] Holden P. A. Klaessig F. Turco R. F. Priester J. H. Rico C. M. Avila-Arias H. (2014a). Evaluation of exposure concentrations used in assessing manufactured nanomaterial environmental hazards: Are they relevant? *Environ. Sci. Technol.* 48 10541–10551. 10.1021/es502440s 25158225

[B25] Holden P. A. Schimel J. P. Godwin H. A. (2014b). Five reasons to use bacteria when assessing manufactured nanomaterial environmental hazards and fates. *Curr. Opin. Biotech.* 27 73–78. 10.1016/j.copbio.2013.11.008 24863899

[B26] Holden P. A. Mortimer M. Wang Y. (2018). Engineered nanomaterials and symbiotic dinitrogen fixation in legumes. *Curr. Opin. Environ. Sci. Health* 6 54–59. 10.1016/j.coesh.2018.07.012

[B27] Holden P. A. Nisbet R. M. Lenihan H. S. Miller R. J. Cherr G. N. Schimel J. P. (2013). Ecological nanotoxicology: integrating nanomaterial hazard considerations across the subcellular, population, community, and ecosystems levels. *Acc. Chem. Res.* 46 813–822. 10.1021/ar300069t 23039211

[B28] Huang W. Tao F. Li F. Mortimer M. Guo L.-H. (2020). Antibacterial nanomaterials for environmental and consumer product applications. *NanoImpact* 20:100268. 10.1016/j.impact.2020.100268

[B29] Ivask A. Juganson K. Bondarenko O. Mortimer M. Aruoja V. Kasemets K. (2014). Mechanisms of toxic action of Ag, ZnO and CuO nanoparticles to selected ecotoxicological test organisms and mammalian cells in vitro: a comparative review. *Nanotoxicology* 8 (Suppl. 1), 57–71. 10.3109/17435390.2013.855831 24256211

[B30] Kiley P. J. Beinert H. (2003). The role of Fe-S proteins in sensing and regulation in bacteria. *Curr. Opin. Microbiol.* 6 181–185. 10.1016/s1369-5274(03)00039-0 12732309

[B31] Le Boulch M. Déhais P. Combes S. Pascal G. (2019). The MACADAM database: a MetAboliC pAthways DAtabase for Microbial taxonomic groups for mining potential metabolic capacities of archaeal and bacterial taxonomic groups. *Database* 2019:baz049. 10.1093/database/baz049 31032842 PMC6487390

[B32] Liao S. Zhang Y. Pan X. Zhu F. Jiang C. Liu Q. (2019). Antibacterial activity and mechanism of silver nanoparticles against multidrug-resistant *Pseudomonas aeruginosa*. *Int. J. Nanomed.* 14 1469–1487. 10.2147/ijn.s191340 30880959 PMC6396885

[B33] Lu J. Struewing I. Buse H. Y. Kou J. Shuman H. A. Faucher S. P. (2013). *Legionella pneumophila* transcriptional response following exposure to CuO nanoparticles. *Appl. Environ. Microbiol.* 79 2713–2720. 10.1128/aem.03462-12 23416998 PMC3623207

[B34] Lu J. Wang Y. Jin M. Yuan Z. Bond P. Guo J. (2019). Both silver ions and silver nanoparticles facilitate the horizontal transfer of plasmid-mediated antibiotic resistance genes. *Water Res.* 169:115229. 10.1016/j.watres.2019.115229 31783256

[B35] Lu T. Qu Q. Lavoie M. Pan X. Peijnenburg W. J. G. M. Zhou Z. (2020). Insights into the transcriptional responses of a microbial community to silver nanoparticles in a freshwater microcosm. *Environ. Pollut.* 258:113727. 10.1016/j.envpol.2019.113727 31838393

[B36] Lu X. Feng X. Werber J. R. Chu C. Zucker I. Kim J.-H. (2017). Enhanced antibacterial activity through the controlled alignment of graphene oxide nanosheets. *Proc. Natl. Acad. Sci. U S A.* 114 E9793–E9801. 10.1073/pnas.1710996114 29078354 PMC5699062

[B37] Luche S. Eymard-Vernain E. Diemer H. Van Dorsselaer A. Rabilloud T. Lelong C. (2016). Zinc oxide induces the stringent response and major reorientations in the central metabolism of *Bacillus subtilis*. *J. Proteomics* 135 170–180. 10.1016/j.jprot.2015.07.018 26211718

[B38] Majumdar S. Keller A. A. (2020). Omics to address the opportunities and challenges of nanotechnology in agriculture. *Crit. Rev. Environ. Sci. Technol.* 2020 1–42. 10.1080/10643389.2020.1785264

[B39] Mauter M. S. Zucker I. Perreault F. Werber J. R. Kim J.-H. Elimelech M. (2018). The role of nanotechnology in tackling global water challenges. *Nat. Sustain.* 1 166–175. 10.1038/s41893-018-0046-8

[B40] McQuillan J. S. Shaw A. M. (2014). Differential gene regulation in the Ag nanoparticle and Ag^+^-induced silver stress response in *Escherichia coli*: A full transcriptomic profile. *Nanotoxicology* 8 177–184. 10.3109/17435390.2013.870243 24392705

[B41] Mock A. Warta R. Dettling S. Brors B. Jäger D. Herold-Mende C. (2018). MetaboDiff: an R package for differential metabolomic analysis. *Bioinformatics* 34 3417–3418. 10.1093/bioinformatics/bty344 29718102 PMC6157071

[B42] Mortimer M. Devarajan N. Li D. Holden P. A. (2018). Multiwall carbon nanotubes induce more pronounced transcriptomic responses in *Pseudomonas aeruginosa* PG201 than graphene, exfoliated boron nitride, or carbon black. *ACS Nano* 12 2728–2740. 10.1021/acsnano.7b08977 29455524

[B43] Mortimer M. Li D. Wang Y. Holden P. A. (2020). Physical properties of carbon nanomaterials and nanoceria affect pathways important to the nodulation competitiveness of the symbiotic N_2_ -fixing bacterium *Bradyrhizobium diazoefficiens*. *Small* 16:e1906055. 10.1002/smll.201906055 31899607

[B44] Mortimer M. Petersen E. J. Buchholz B. A. Orias E. Holden P. A. (2016). Bioaccumulation of multiwall carbon nanotubes in *Tetrahymena thermophila* by direct feeding or trophic transfer. *Environ. Sci. Technol.* 50 8876–8885. 10.1021/acs.est.6b01916 27398725 PMC4991038

[B45] Mu D. S. Yu X. X. Xu Z. X. Du Z. J. Chen G. J. (2016). Physiological and transcriptomic analyses reveal mechanistic insight into the adaption of marine *Bacillus subtilis* C01 to alumina nanoparticles. *Sci. Rep.* 6:10. 10.1038/srep29953 27440502 PMC4954987

[B46] Mu L. Zhou Q. X. Zhao Y. J. Liu X. W. Hu X. G. (2019). Graphene oxide quantum dots stimulate indigenous bacteria to remove oil contamination. *J. Hazard. Mater.* 366 694–702. 10.1016/j.jhazmat.2018.12.044 30583239

[B47] Ouyang K. Mortimer M. Holden P. A. Cai P. Wu Y. Gao C. (2020). Towards a better understanding of *Pseudomonas putida* biofilm formation in the presence of ZnO nanoparticles (NPs): Role of NP concentration. *Environ. Int.* 137:105485. 10.1016/j.envint.2020.105485 32004708

[B48] Park S. B. Steadman C. S. Chaudhari A. A. Pillai S. R. Singh S. R. Ryan P. L. (2018). Proteomic analysis of antimicrobial effects of pegylated silver coated carbon nanotubes in *Salmonella enterica* serovar *Typhimurium*. *J. Nanobiotechnol.* 16:31. 10.1186/s12951-018-0355-0 29587743 PMC5870919

[B49] Peng M. W. Yu X. L. Guan Y. Liu P. Yan P. Fang F. (2019). Underlying promotion mechanism of high concentration of silver nanoparticles on anammox process. *ACS Nano* 13 14500–14510. 10.1021/acsnano.9b08263 31794189

[B50] Planchon M. Leger T. Spalla O. Huber G. Ferrari R. (2017). Metabolomic and proteomic investigations of impacts of titanium dioxide nanoparticles on *Escherichia coli*. *PLoS One* 12:e0178437. 10.1371/journal.pone.0178437 28570583 PMC5453534

[B51] Poirier I. Kuhn L. Demortiere A. Mirvaux B. Hammann P. Chicher J. (2016). Ability of the marine bacterium *Pseudomonas fluorescens* BA3SM1 to counteract the toxicity of CdSe nanoparticles. *J. Proteomics* 148 213–227. 10.1016/j.jprot.2016.07.021 27523480

[B52] Priester J. H. Stoimenov P. K. Mielke R. E. Webb S. M. Ehrhardt C. Zhang J. P. (2009). Effects of soluble cadmium salts versus CdSe quantum dots on the growth of planktonic *Pseudomonas aeruginosa*. *Environ. Sci. Technol.* 43 2589–2594. 10.1021/es802806n 19452921

[B53] Qian H. F. Zhu K. Lu H. P. Lavoie M. Chen S. Zhou Z. J. (2016). Contrasting silver nanoparticle toxicity and detoxification strategies in *Microcystis aeruginosa* and *Chlorella vulgaris*: New insights from proteomic and physiological analyses. *Sci. Total Environ.* 572 1213–1221. 10.1016/j.scitotenv.2016.08.039 27522289

[B54] Ren B. Wang X. Duan J. Ma J. (2019a). Rhizobial tRNA-derived small RNAs are signal molecules regulating plant nodulation. *Science* 365 919–922. 10.1126/science.aav8907 31346137

[B55] Ren W. Ding Y. Gu L. Yan W. Wang C. Lyu M. (2019b). Characterization and mechanism of the effects of Mg-Fe layered double hydroxide nanoparticles on a marine bacterium: new insights from genomic and transcriptional analyses. *Biotechnol. Biofuels* 12:196. 10.1186/s13068-019-1528-2 31428192 PMC6696678

[B56] Revel M. Châtel A. Mouneyrac C. (2017). Omics tools: New challenges in aquatic nanotoxicology? *Aquat. Toxicol.* 193 72–85. 10.1016/j.aquatox.2017.10.005 29049925

[B57] Ryan D. Jenniches L. Reichardt S. Barquist L. Westermann A. J. (2020). A high-resolution transcriptome map identifies small RNA regulation of metabolism in the gut microbe *Bacteroides thetaiotaomicron*. *Nat. Commun.* 11 3557–3557. 10.1038/s41467-020-17348-5 32678091 PMC7366714

[B58] Sohm B. Immel F. Bauda P. Pagnout C. (2015). Insight into the primary mode of action of TiO_2_ nanoparticles on *Escherichia coli* in the dark. *Proteomics* 15 98–113. 10.1002/pmic.201400101 25346333

[B59] Song Z. H. Niu C. Wu H. Wei J. P. Zhang Y. X. Yue T. L. (2019). Transcriptomic analysis of the molecular mechanisms underlying the antibacterial activity of IONPs@pDA-nisin composites toward *Alicyclobacillus acidoterrestris*. *ACS Appl. Mater. Interfaces* 11 21874–21886. 10.1021/acsami.9b02990 31185568

[B60] Su Y. Zheng X. Chen Y. Li M. Liu K. (2015). Alteration of intracellular protein expressions as a key mechanism of the deterioration of bacterial denitrification caused by copper oxide nanoparticles. *Sci. Rep.* 5:15824. 10.1038/srep15824 26508362 PMC4623765

[B61] Vernis L. El Banna N. Baïlle D. Hatem E. Heneman A. Huang M.-E. (2017). Fe-S clusters emerging as targets of therapeutic drugs. *Oxid. Med. Cell. Longev.* 2017 3647657–3647657. 10.1155/2017/3647657 29445445 PMC5763138

[B62] Vicente A. Sohm B. Flayac J. Rousselle P. Bauda P. Pagnout C. (2019). Toxicity mechanisms of ZnO UV-filters used in sunscreens toward the model cyanobacteria *Synechococcus elongatus* PCC 7942. *Environ. Sci. Pollut. Res. Int.* 26 22450–22463. 10.1007/s11356-019-05057-6 31161548

[B63] Wang X. Lee J. H. Li R. Liao Y. P. Kang J. Chang C. H. (2018a). Toxicological profiling of highly purified single-walled carbon nanotubes with different lengths in the rodent lung and *Escherichia coli*. *Small* 14:e1703915. 10.1002/smll.201703915 29733549 PMC6239219

[B64] Wang X. Mansukhani N. D. Guiney L. M. Lee J. H. Li R. B. Sun B. B. (2016). Toxicological profiling of highly purified metallic and semiconducting single-walled carbon nanotubes in the rodent lung and *E. coli*. *ACS Nano* 10 6008–6019. 10.1021/acsnano.6b01560 27159184 PMC4941827

[B65] Wang Y. Chang C. H. Ji Z. X. Bouchard D. C. Nisbet R. M. Schimel J. P. (2017). Agglomeration determines effects of carbonaceous nanomaterials on soybean nodulation, dinitrogen fixation potential, and growth in soil. *ACS Nano* 11 5753–5765. 10.1021/acsnano.7b01337 28549216 PMC5860665

[B66] Wang Y. Mortimer M. Chang C. H. Holden P. A. (2018b). Alginic acid-aided dispersion of carbon nanotubes, graphene, and boron nitride nanomaterials for microbial toxicity testing. *Nanomaterials* 8:76. 10.3390/nano8020076 29385723 PMC5853708

[B67] Wen B. Mei Z. Zeng C. Liu S. (2017). metaX: a flexible and comprehensive software for processing metabolomics data. *BMC Bioinformatics* 18:183. 10.1186/s12859-017-1579-y 28327092 PMC5361702

[B68] Werlin R. Priester J. H. Mielke R. E. Kramer S. Jackson S. Stoimenov P. K. (2011). Biomagnification of cadmium selenide quantum dots in a simple experimental microbial food chain. *Nat. Nanotechnol.* 6 65–71. 10.1038/nnano.2010.251 21170041

[B69] Westmeier D. Hahlbrock A. Reinhardt C. Frohlich-Nowoisky J. Wessler S. Vallet C. (2018). Nanomaterial-microbe cross-talk: physicochemical principles and (patho)biological consequences. *Chem. Soc. Rev.* 47 5312–5337. 10.1039/c6cs00691d 29770369

[B70] Xu F. F. Imlay J. A. (2012). Silver(I), mercury(II), cadmium(II), and zinc(II) target exposed enzymic iron-sulfur clusters when they toxify *Escherichia coli*. *Appl. Environ. Microbiol.* 78 3614–3621. 10.1128/aem.07368-11 22344668 PMC3346352

[B71] Yan X. T. He B. Liu L. H. Qu G. B. Shi J. B. Hu L. (2018). Antibacterial mechanism of silver nanoparticles in *Pseudomonas aeruginosa*: proteomics approach. *Metallomics* 10 557–564. 10.1039/c7mt00328e 29637212

[B72] Yan X. Song M. Zhou M. Ding C. Wang Z. Wang Y. (2019). Response of *Cupriavidus basilensis* B-8 to CuO nanoparticles enhances Cr(VI) reduction. *Sci. Total Environ.* 688 46–55. 10.1016/j.scitotenv.2019.05.438 31229827

[B73] Zhang J. L. Zhou Z. P. Pei Y. Xiang Q. Q. Chang X. X. Ling J. (2018). Metabolic profiling of silver nanoparticle toxicity in *Microcystis aeruginosa*. *Environ. Sci. Nano* 5 2519–2530. 10.1039/c8en00738a

[B74] Zhang Y. R. Mortimer M. Guo L. H. (2020). Interplay between engineered nanomaterials and microbiota. *Environ. Sci. Nano* 7 2454–2485. 10.1039/d0en00557f

[B75] Zhao C. Wu L. Wang X. Weng S. Ruan Z. Liu Q. (2020). Quaternary ammonium carbon quantum dots as an antimicrobial agent against gram-positive bacteria for the treatment of MRSA-infected pneumonia in mice. *Carbon* 163 70–84. 10.1016/j.carbon.2020.03.009

[B76] Zheng X. Wang J. Chen Y. G. Wei Y. Y. (2018). Comprehensive analysis of transcriptional and proteomic profiling reveals silver nanoparticles-induced toxicity to bacterial denitrification. *J. Hazard. Mater.* 344 291–298. 10.1016/j.jhazmat.2017.10.028 29055833

[B77] Zhu Y. Orre L. M. Zhou Tran Y. Mermelekas G. Johansson H. J. Malyutina A. (2020). DEqMS: A method for accurate variance estimation in differential protein expression analysis. *Mol. Cell. Proteomics* 19 1047–1057. 10.1074/mcp.tir119.001646 32205417 PMC7261819

